# The Role of Nursing Informatics on Promoting Quality of Health Care and the Need for Appropriate Education

**DOI:** 10.5539/gjhs.v6n6p11

**Published:** 2014-06-24

**Authors:** Asieh Darvish, Fatemeh Bahramnezhad, Sara Keyhanian, Mojdeh Navidhamidi

**Affiliations:** 1 School of Nursing and Midwifery, Tehran University of Medical Sciences, Tehran, Iran; 2 School of Allied Medical Sciences, Tehran University of Medical Sciences, Tehran, Iran; 3 Electrophysiology Research Center, Neuroscience Institute, Tehran University of Medical Sciences, Tehran, Iran

**Keywords:** education, nursing, quality of health care, nursing informatics, technology

## Abstract

In today’s dynamic health systems, technology plays an important role in education and nursing work. So it seems necessary to study the role of nurses and highlight the need for appropriate information technology educational programs to integrate with the ever-increasing pace of technology. A review accompanied by an extensive literature search in databases and a library search focused on the keywords were used. The criteria used for selecting studies primarily focused on nursing informatics and the importance of expertise in the effective use of information technology in all aspects of the nursing profession. In a critical assessment of emerging technologies, the key elements of nursing informatics implementation were considered as healthcare promotion, advanced systems, internet and network. In view of the nature and the development of the information age, it is required to receive necessary IT training for all categories of nurses. Due to the fast development of technology, in order to effectively take advantage of information technology in nursing outcome and quality of health care and to empower nurses; educational arrangement is recommended to set short-term and long-term specialized courses focusing on four target groups: studying, working, graduate, senior undergraduate, and graduate doctoral. The result of this study is expected to assist educational providers with program development.

## 1. Introduction

### 1.1 History and Definition

Nurses has been working in the field of informatics near four decades, the term “nursing informatics” has been considered a specialization in nursing resources since 1984 (Guenther & Peters, 2006). Many aspects such as data recovery, ethics, patient care, decision support systems, human-computer interaction, information systems, imaging informatics, computer science, information science, security, electronic patient records, intelligent systems, e-learning and telenursing have been added to the field. Hana has defined Nursing Informatics as the application of IT in the nursing duties including education, management & practice in 1985. Integration of information science, computer science and nursing science to support nursing practice and knowledge management was the definition offered in 1989 by Graves and Corcoran. The American Nurses Association (ANA) published its aim and standards in 1994-1995 and presented the Nursing Informatics as a specialty that integrates nursing science, computer and information science to provide data communication management, knowledge and nursing work in 2001. Now most of nursing professionals believe that it is defined as the integration of information technology and all aspects of nursing such as clinical nursing, management, research or education (Guenther & Peters, 2006).

### 1.2 Competencies

The competency of nursing informatics specialists was determined through studying three categories including computer skills, informatics knowledge and informatics skills. It investigates four levels of nursing practice: beginning nurse, experienced nurse, informatics specialist, and informatics innovator.

The following competencies were rejected: diagnostic coding, desktop publishing, managing central facilities to enable data sharing and writing an original computer program (Staggers et al., 2002). Some components of accepted competencies are shown below in brief.

#### 1.2.1 Computer Skills

Selected computer skill competencies contain computerized searches and retrieving patient demographics data, the use of telecommunication devices, the documentation of patient care, the use of information technologies for improving nursing care, and the use of networks and computer technology safely.

#### 1.2.2 Informatics Knowledge

Selected informatics knowledge competencies are the recognition of the use or importance of nursing data for improving practice, and the recognition of the fact that the computer can only facilitate nursing care and that there are human functions that cannot be performed by computers, the formulation of ethical decisions in computing, the recognition of the value of clinicians’ involvement in the design, selection, implementation, and evaluation of systems in health care, the description of the present manual systems, the definition of the impact of computerized information management on the role of the nurse and the determination of the limitations and the reliability of computerized patient monitoring systems.

#### 1.2.3 Informatics Skills

Informatics skills competencies includes the interpretation of information flow within the organization, the preparation of process information flow charts for all aspects of clinical systems, the development of standards and database structures to facilitate clinical care, education, administration or research. It also includes the development of innovative and analytic techniques for scientific inquiry in nursing informatics and new data organizing methods and research designs with the aim of examining the impacts of computer technology on nursing, and the conducting of basic science research to support the theoretical development of informatics. Information literacy skills, competencies, and knowledge are investigated among educators, administrators and clinicians of nursing groups nationally.

### 1.3 The Importance of Nursing Informatics

The history, definition and competencies of nursing informatics indicate the importance of this field. It shows nurses are integrated into the field of IT automatically. So they should be able to deal with it successfully to improve quality of care outcome. In this regard it is required to study the influence of nursing informatics on health care and make bold the appropriate information technology educational needs for nurses.

## 2. Method

An extensive literature search was performed by using databases Pubmed, Google Scholar, Ovid, Science Direct and SID. Search terms were “education, nursing”; “quality of health care”; “nursing informatics” and technology. The study was carried out from January to April, 2014. A library search was also performed. As many as 135 articles were retrieved. With a critical point of view, 40 articles in English were selected that specifically focused on nursing informatics education and its influence on nursing outcomes and the quality of health care (Staggers et al., 2002).

## 3. Results

The study mentions the followings as the key elements of nursing informatics implementation:

### 3.1 Health Care Promotion

The advantages of applying information technology in all aspects of nursing, including clinical areas, management, education and research and its influence on health care have been reviewed. Today, the subjects of clinical nursing information systems, decision support systems and medical diagnostic systems are associated with collecting patient information. Regarding the technology-rich environment, health care and hospital information systems developers, the quality of care is improving. For increasing patient safety and its leading to an evidence-based nursing, nursing informatics has been enhanced for students and graduates by Columbia school of nursing. The study has proved that informatics competence is a prerequisite to improving patient care (Bakken et al., 2003). Technology and using multimedia integrated into nursing curriculum can promote the use of informatics tools as an integral practice component and increase patient safety (Norton et al., 2006). Managers can improve efficiency and performance through information systems and new technologies. In addition, several studies have confirmed the impact of careful shift planning and efficient management on nurse’s work and the quality of health care. Information is the source of all management activities. Nursing care is an industry service and its product is patient care. Information technology can promote the nursing management outcome. Internet-based nurse scheduling systems are mostly designed according to the self-scheduling model and need refining by the manager who overviews proper distribution, it causes uniform resource allocation in scheduling and increases patient direct care time (Pierce et al., 2003). Implementing information systems can provide better access to evidence; it can affect the patient care quality and support evidence-based nursing. Software tools to facilitate research are available in all medical fields (Kardan & Darvish, 2008). Nursing information system had an influence on clinical patterns and decreased the time nurses spent on indirect care (Darvish & Salsali, 2010). This is critical to the health care professionals to assess, apply, report and manage data by the help of new tools of the information age (Hall, 1995).

### 3.2 Advanced Systems

Although using decision support systems can lead to a safer care, it may impair critical thinking. The need for excessive working time could cause some delay in the nursing job and reduce the quality of health care (Norton, 2006). Researchers recommended considering the following:

-Involving nurse managers in the system selection and designing process
-Designing a simple and efficient process
-Recording a system-compatible guideline
-Improving the system speed
-Selecting hardware which can encourage nurses to use them
-Upgrading the system through innovation in information technology
Decision support systems have been defined to assist physicians to solve problems that require specific decisions since 30 years ago. It is replacing the role of human knowledge by formulating the knowledge in the system (Ting et al., 2008). Expert systems are the most common types of clinical decision support systems and have applications in show notes, diagnostic support, critical treatment plans, decision support, prescriptions, recovery and the identification and the interpretation of pictures, however, as stand-alone tools, are not able to replace human expertise. These systems should be integrated with knowledge management. Several studies have shown that the integration of implicit and explicit knowledge and management of different types of knowledge will help to determine the best treatment plan. Logical design is required for the success of these systems and seems to hardly have been considered. The application of guides and easy access to up-to-date clinical evidence and the cutting of duplicate tests could reduce medical mistakes and improve quality of care, but there are some limitations (Montani & Bellazzi, 2002). To increase the usefulness and acceptability of such systems, the ease of use, support and maintenance combined with the ability of systems hardware, software applications, integration with hospital information systems and patient records should be considered (Holbrook et al., 2003). Including smart and intelligent tools in diagnosis and treatment methods can reduce medical errors and harm as well as financial loss for humans. Artificial intelligence and expert systems are used to help the diagnosis.

### 3.3 Internet and Network

The first internet-based Nursing Informatics courses were offered by Duck University in 1997. Represented advantages were clearly defined and measurable learning outcomes and real-world problems were introduced as the main component of instructional strategies. There were some disadvantages like hardware and software problems, deficiency in prerequisite skills, troubleshooting difficulties, and low internet access speed and poor time management to master the material (Goodwin, 1997). The evaluation of an innovative consumer health informatics intervention proved that the patient and nurses are satisfied with the use of electronic and communication devices and home care (Kossman et al., 2006). Nurses can have networks such as AJN and AMN now. In the world wide web, it is possible to have a common network for nursing organizations and develop a forum to discuss nursing issues and design online meetings (Rizzolo & DuBois, 1995). The other aspects of nursing practice potential in the new decade are offering services from distance through telemedicine or telenursing. In this regard, easy remote diagnostic software and hardware are designed to facilitate E-health services. Tele-nurses can provide various services such as education, patient monitoring and counselling through Internet facilities. Telenurses are satisfied with their role. They care remotely using special skills and knowledge. It can cover nurse shortages and the global demand for nurses (Darvish & Salsali, 2010). In a telenursing Survey most of nurses believed that it is better to design educational programs for nurses to be able to work as telenurse (Grady, 2007). Using online library resources and outreach programs would be beneficial and produce positive outcomes for nurses (Wozar & Worona, 2003). Information and communication technology progress provided the possibility of improving health through e-education irrespective of time and place. Patient education systems on the internet can increase patient satisfaction and influence their self-care behaviour. E-health educational programs make people aware of disease management and increase coordination with the health care professional team. It influences the life style and the prevention of diseases such as cancer, HIV and chronic diseases. On the other hand, it empowers medical groups by enhancing and upgrading their knowledge. The web-based computer simulation educational program in crisis decreased medical errors in emergency departments. Information technology application refers to providing simultaneous access to education in specified locations which require huge spending. It decreases cost loss (Kardan & Darvish, 2007). Online access to journal articles prevents repeated research programs and makes assessment easy. Online databases provided up-to-date article access and informed nurses about new technologies, easy software and the results of investigations (Darvish, 2010).

### 3.4 Related Organizations Activity

National advisory council on nurse education and practice addresses nursing practice challenges; and mentions electronic health records, patient monitoring systems, bar code medication administration, computerised provider order entry, data capture tools, care planning tools and telehealth; and the need to consider nursing shortage, reducing medical errors, improve tracking of patient data, improve efficiency of data collection, improve access to care, support national surrveilance capabillities. These are also mentioned as ways to address nursing education challenges: E-learning to deliver education, simulation to deliver education, inclusion of Health Care IT in curricula, and faculty development for health care. It centeralizes these as solutions which can improve capacity of nursing schools, reduce faculty shortage, and increase health IT skills of graduating students. There are many group projects and organizations which support nurses’ involvment for optimal use of IT in their job. Some are introduced in the following:

-The National Coordinator for Health Information Technology (ONC)
-The American Health Information Community (AHIC)
-The Nationwide Health Information Network (NHIN)
-The Faculty Development: Integrated Technology into Nursing Education and Practice Initiative (ITNEP)
-National Advisory Council on Nurse Education and Practice (NACNEP)
-National League of Nursing (NLN)
-National Institute of Health (NIH)
-National Institute of Nursing Research (NINR)
-North American Nursing Diagnosis International (NANDA)
-Technology Informatics Guiding Education Reform Initiative (TIGER)

It is reported that without proper training for nurses, efforts to integrate healthcare IT with nursing practice will be hampered. It gives evidence that nurses are not getting adequate training for IT usage (NACNEP annual report, 2009).

### 3.5 Need for Educational Programs

Due to the fast growth and development of technology, in order to effectively take advantage of information technology in nursing outcome and quality of health care, educational arrangement is recommended to strengthen nurses at different levels for implementing information technology tools in all aspects of their profession.

## 4. Conclusion

In today’s world the potential for information and communication technology application is increasing so that it can enhance the quality of nursing domains outcome (McNelis et al., 2012). Nurses have the most communication with patients, and interact with technology more frequently. Using technology should create a positive attitude in nursing productivity. It is essential for nurses to be involved in the initial design of systems to improve the quality of health care and change their culture in this regard (Darvish & Salsali, 2010), (Jenkins et al., 2007).

**Figure 1:**
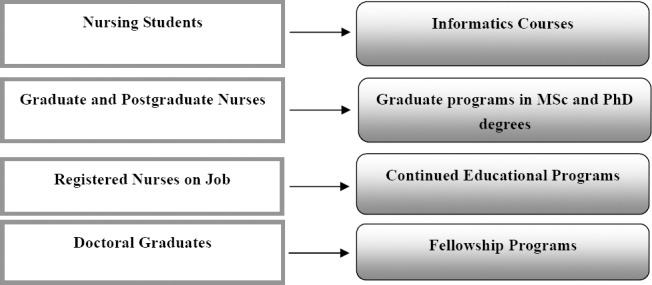
The proposed educational model for empowering nurses on the subject of nursing informatics in four groups

Mediating technically and technologically on the borderline between medicine and nursing, nurses have become known as the medical Goddesses in the form of Tele-nurses. Nurses have got more authority in decision-making with the use of new technologies (Gassert, 1998). For successful implementation of the electronic health reporting system, nurses must be knowledgeable about information technology, computer skills and informatics knowledge and skills. In telenursing, the importance of data quality criteria, transparency and integrity, authenticity, confidentiality, the updating of information, accountability, productivity, standards and accessibility of health web sites should be considered (Darvish, 2008). The NACNEP recommended to prepare nurses to adopt intelligent and quality-based information technology use in patient care by implementing five strategies: providing core informatics courses to nursing schools, educating nurses specialized in informatics skills who are able to solve related issues, offering more powerful nursing care through the implication of telecommunication projects, preparing more nursing faculties in the informatics field to facilitate students skills enhancement and enhancing collaboration to advance informatics. The benefits of extending nursing informatics strategies directly and indirectly influence patient and people health positively (Matarrese & Helwig, 2000). Courses affect nursing students’ perceptions about informatics (Jetté et al., 2010); and they may learn at the BSc level about patient-centered evidence-based care through the use of informatics tools, and get informed about benefits such as promotion of safety, quality and effective clinical decisions (Norton et al., 2006; Ainsley & Brown, 2009). The may even learn how remote care and personal phone can improve nursing care in different areas such as psychiatric nursing (Tseng et al., 2012; Goossen, 2007; Wittmann-Price, 2012). At the same time, nursing workers are busy in the wards giving care. If they are not alert to new technologies, it will be difficult to accept the new nurses’ ideas who are educated recently with a positive attitude to the advantages of information technology. This group of nurses can be encouraged to be integrated into the potential of E-learning as well as continuing education (Button, 2013), based on the summit of technology informatics guide education reform (Schlak & Troseth, 2013). It seems necessary to prepare knowledgeable nurses to deal with selecting, developing, implementing and evaluating IT to interpret data as usable knowledge and information. In the nursing world, four domains should be empowered. Undergraduate and diploma programs can be integrated with courses. Graduate programs can be designed. Formal and informal continued educational programs for nurses on job and fellowships for PhD graduated nurses can be useful. Trying to make different groups of nurses ready for the ever-increasing speed of technology in the current century is possible, not only by parallel opportunities of learning, but also with the help of evaluating tools such as Self-assessment of nursing informatics competencies scale which can bring the same range of comprehention about informatics implementation (Choi & Bakken, 2013). In conclusion, considering nursing outcomes takes advantage of information technology; educational arrangement is recommended to set short-term and long-term specialized courses focusing on the four target groups. Informatics courses for nursing students continued educational programs for registered nurses in work area, graduate programs at MSc and PhD levels for nurses and fellowship programs for doctoral graduates are recommended to be considered (Figure 1).

It is essential that nurse educators incorporate the entire concept of informatics into the education of nurses.
